# Anti-shivering Drug Influences the Characteristics of Electroencephalographic Shivering Noise During Targeted Temperature Management: A Case Report

**DOI:** 10.7759/cureus.84509

**Published:** 2025-05-20

**Authors:** Hiroyuki Honda, Kei Nishiyama

**Affiliations:** 1 Department of Critical Care Medicine, Niigata University Medical and Dental Hospital, Niigata, JPN; 2 Division of Emergency and Critical Care, Niigata University School of Medicine, Niigata, JPN

**Keywords:** artifact, cardiac arrest, electroencephalography, shivering, targeted temperature management

## Abstract

We report a case of a 75-year-old male patient who underwent targeted temperature management (TTM) following cardiopulmonary resuscitation and subsequently experienced uncontrolled shivering. Rocuronium was administered to alleviate shivering; however, the shivering recurred, necessitating continuous administration of dexmedetomidine. Analysis of the electroencephalography (EEG) recorded for neuromonitoring demonstrated that both pharmacological agents significantly reduced the power of the alpha and beta waves at 10 and 60 minutes post-administration. Dexmedetomidine also influenced the delta and theta waves, coinciding with the cessation of shivering, whereas muscle relaxants exhibited minimal effects on these frequency bands. These findings suggest that shivering-induced noise causing EEG changes is drug-dependent and predominantly affects high-frequency regions, potentially impacting the neurological prognosis predictions and detection of nonconvulsive status epilepticus (NCSE). This case illustrates the need to recognize the effects of shivering and anti-shivering medications on EEG monitoring during post-resuscitation care. Further research is warranted to evaluate the impact of these factors on EEG-based prognostication and NCSE detection, and to optimize strategies for managing shivering-induced artifacts during TTM.

## Introduction

Targeted temperature management (TTM) is a fundamental aspect of post-cardiac arrest care for patients who remain unresponsive following the return of spontaneous circulation [1]. This intervention involves regulating the patient's body temperature within a specified range to confer neuroprotection and enhance neurological outcomes. As part of this care, electroencephalography (EEG) is utilized to predict neurological prognosis and identify nonconvulsive status epilepticus (NCSE) [2,3]. However, shivering, a result of thermoregulatory processes, introduces artifacts into the EEG, complicating its interpretation [4]. During episodes of shivering, the electrical activity of the brain is obscured by superimposed myoelectric activity, thereby distorting the frequency analysis results and rendering neurological prognostication unfeasible. This interference also hinders the diagnosis of NCSE, in which epileptic activity must be detected in the EEG in the absence of physical convulsions. The impact of shivering during TTM on EEG and the effects of anti-shivering medications on EEG remain insufficiently elucidated, with limited research available in this domain [5]. Clarifying these effects could significantly contribute to optimizing post-resuscitation care.

Herein, we encountered a scenario in which pharmacological management of shivering occurred during TTM, and we were able to document a sustained forehead EEG of these effects. The present report underscores the importance of prudence in EEG analysis within the framework of post-resuscitation care. To this end, the effects of shivering and anti-shivering drugs on EEG over time were demonstrated, along with the results of the frequency analysis.

## Case presentation

Written informed consent was obtained from the patient for publication of this report. A 75-year-old man, 155 cm tall and weighing 40 kg, was admitted for thorough examination of recurrent fainting episodes. The patient experienced cardiopulmonary arrest resulting from ventricular fibrillation in the general ward. Cardiopulmonary resuscitation was promptly initiated, and spontaneous circulation was restored after 8 minutes. However, the patient remained comatose and was subsequently transferred to the intensive care unit for further treatment. He was intubated and underwent TTM using a body surface cooling device (ArcticSun, Medivance, Inc., Louisville, CO) that featured a temperature feedback mechanism. According to the TTM protocol employed at our institution, the target core body temperature was set at 36°C and maintained for 24 hours, followed by a 10-hour period of gradual warming up to 37°C at a rate of 0.1℃/hour, and finally maintained at 37°C for an additional 26 hours.

The patient received midazolam, 0.25 mg/kg/hour, and fentanyl, 0.6 µg/kg/hour, for sedation and analgesia, and noradrenaline (up to 0.3 µg/kg/minute), for circulatory failure. Shivering was assessed using the Bedside Shivering Assessment Scale (BSAS) [6]. To prevent shivering, counterwarming measures were administered along with intravenous acetaminophen. Continuous two-channel forehead EEG was conducted to evaluate the presence of epileptic activity. Approximately 23 hours after TTM initiation, BSAS 1 shivering was observed. Concurrently, noise was introduced into the EEG. Despite an increase in the fentanyl dosage to 1.1 µg/kg/hour, shivering persisted, necessitating the administration of a 50 mg bolus of rocuronium. This immediately ceased shivering; however, BSAS 1 shivering recurred approximately 6 hours after muscle relaxant administration. In response, dexmedetomidine was administered as a continuous intravenous infusion started at 0.7 µg/kg/hour. Finally, the shivering resolved and did not recur, allowing TTM to be performed as planned. The findings indicated the absence of epileptic EEG patterns throughout the TTM period. Following TTM completion, the patient regained consciousness and was successfully extubated. On the eighth day of ICU stay, he was transferred to the general ward.

EEG analysis

The EEG was measured at the forehead (Fp1 and Fp2, as defined by the international 10-20 method) using two-channel EEG measurement modules (IntelliVue EEG module, Philips Medizin Systeme Böblingen GmbH, Germany) integrated into a bedside monitor (IntelliVue, Philips Medizin Systeme). Their reference electrodes were attached to the earlobes (A1 and A2, respectively), and the ground electrode was centered on the forehead. The EEG data were filtered with a 0.5 Hz high-pass and 30 Hz low-pass filter and stored on a dedicated server at a 125 Hz sampling rate. EEG data containing artifacts that could compromise the analysis through visual inspection were excluded. Sequential EEG data were divided into 5-minute epochs, from which 50 samples of 10-second EEGs were randomly extracted with replacement from each of Fp1 and Fp2. EEG features were calculated for each of the 100 samples per epoch and averaged to represent that epoch. We employed the Fourier transform to decompose the EEG signals into their constituent frequency components, and subsequently computed the power spectra. EEG power spectra represent the brain's electrical activity distribution across frequency bands, quantified by the signal power at each frequency in microvolts squared per hertz (µV²/Hz). The power spectra for the alpha (8-13 Hz), beta (13-30 Hz), delta (0.5-4 Hz), and theta (4-8 Hz) bands were computed. To evaluate the impact of the administered drug on the EEG, EEG data from 10 minutes before (20-10 minutes before), 10 minutes after (10-20 minutes after), and 60 minutes after (60-70 minutes after) drug administration were compared. In other words, a comparison was made between 200 EEG samples, each comprising 10-minute intervals.

**Figure 1:**
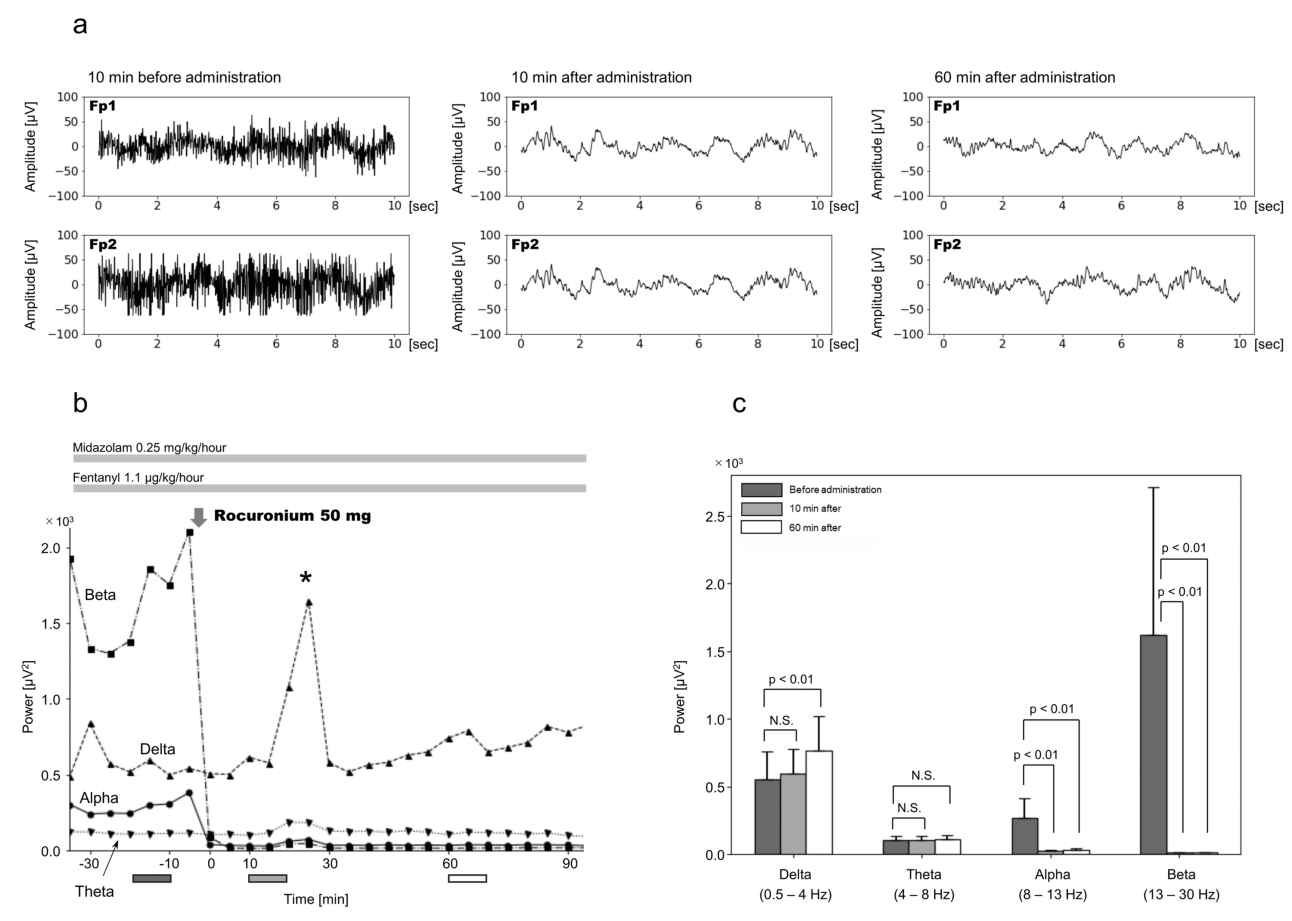
Electroencephalography before and after rocuronium administration The administration of rocuronium for the treatment of shivering resulted in the cessation of shivering, accompanied by a rapid decrease in alpha- and beta-wave band powers. (a) Representative electroencephalography (EEG), 10 minutes before, 10 minutes after, and 60 minutes after administration of rocuronium. (b) Changes in averaged 5-minute alpha-, beta-, theta-, and delta-wave EEG band power from 30 minutes before to 90 minutes after rocuronium administration. (c) Mean EEG band power 10 minutes before rocuronium administration is compared with that 10 and 60 minutes after administration. Each error bar represents the standard deviation. The timing of the measurements, 20-10 minutes before rocuronium administration, from 10-20 minutes, and 60-70 minutes after administration, are indicated by the dark gray, gray, and white boxes below the horizontal axis (a). The asterisk (*) denotes artifacts caused by nursing care. N.S., not significant.

**Figure 2:**
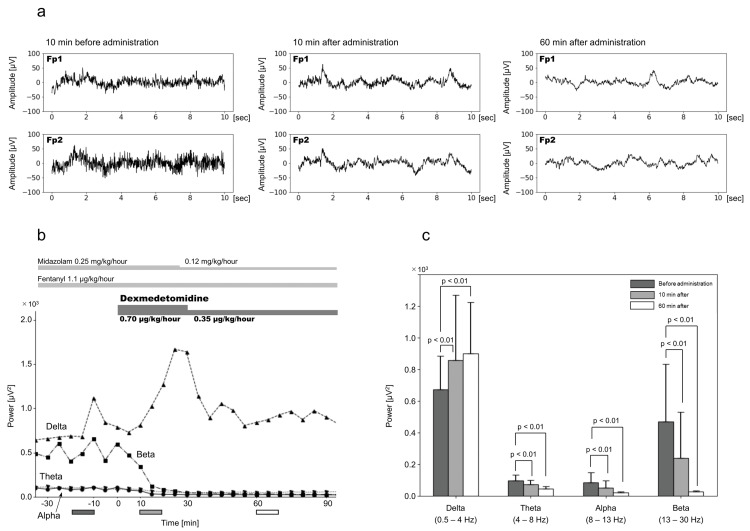
Electroencephalography before and after dexmedetomidine administration Upon continuous administration of dexmedetomidine for management of recurrent shivering, gradual cessation of shivering was observed. Electroencephalography (EEG) indicated a progressive reduction in the power of alpha-, beta-, and theta-wave bands, accompanied by an increase in delta-wave band power. (a) Representative EEG, 10 minutes before, 10 minutes after, and 60 minutes after administration of dexmedetomidine. (b) Changes in averaged 5-minute alpha-, beta-, theta-, and delta-wave EEG band power from 30 minutes before to 90 minutes after dexmedetomidine administration. (c) Mean EEG band power 10 minutes before dexmedetomidine administration is compared with that 10 and 60 minutes after administration. Each error bar represents the standard deviation. The timing of the measurements, from 20-10 minutes before muscle relaxant administration, from 10-0 minutes, and from 60-70 minutes after administration, are indicated by the dark gray, gray, and white boxes below the horizontal axis (a).

Following muscle relaxant administration, alpha- and beta-wave band powers significantly decreased at 10 and 60 minutes, respectively. The delta- and theta-wave band powers at 10 minutes showed no significant changes; however, the delta-wave band power increased significantly at 60 minutes (Figure 1). Dexmedetomidine notably reduced the alpha- and beta-wave band power at both 10 and 60 minutes. In the slow-wave region, the theta-wave band power was significantly decreased, whereas the delta-wave band power significantly increased at both 10 and 60 minutes (Figure 2).

Continuous variables were reported as mean ± standard deviation, and categorical variables as proportions. Welch's t-test compared means at p<0.01. EEG and statistical analyses used Python (v3.8.11; Python Software Foundation, Wilmington, DE). The packages used were Numpy ver. 1.24.3, matplotlib ver. 3.4.2, pandas ver. 1.5.3, SciPy ver. 1.10.1, and scikit-learn ver. 0.24.2. Sample size for Welch's t-test (effect size 0.50, alpha 0.01, power 0.99) was calculated using G*Power 3.1.9.7 (Heinrich Heine University, Düsseldorf, Germany), indicating 194 data points needed for pre- and post-comparisons.

## Discussion

The present case revealed that administering muscle relaxants and dexmedetomidine to suppress shivering produced noticeable EEG changes. Specifically, the muscle relaxants displayed a pronounced decrease in the power spectrum of the alpha- and beta-wave regions, with minimal impact on the slow-wave region. Similarly, dexmedetomidine demonstrated a reduction in the power spectrum of alpha- and beta-wave regions; however, this was accompanied by a decline in the theta-wave power spectrum and an increase in the delta-wave power spectrum. These observations coincided with the absence of shivering, indicating that shivering substantially influences the high-frequency EEG region, while the low-frequency region appears contingent on the therapeutic agent used.

Muscle relaxants are thought to have no influence on the central nervous system. Therefore, the substantial decrease in the alpha- and beta-wave power spectra that occurred when shivering ceased suggests that EEG noise caused by shivering occurs in these frequency bands. The fact that continuous administration of dexmedetomidine decreased the power spectra of the alpha and beta waves and suppressed shivering supports this idea. The electromyographic frequency of shivering is influenced by body weight, using the equation log Y (Hz) = -0.18 log X (g) + 1.89, where Y represents frequency and X represents body weight [7]. According to the formula used, the body weight of the patient (40 kg) corresponded to a frequency of 11.5 Hz, which is within the alpha-wave range. Additionally, the electromyographic frequency of shivering can reach 200 Hz [8]. Therefore, shivering-induced EEG noise is considered to occur in the alpha- and beta-wave regions, which is consistent with the course of the present case.

Dexmedetomidine is deemed to suppress shivering [9,10]. Spindle (9-15 Hz) and slow delta oscillations (0-4 Hz) have been observed in EEGs during dexmedetomidine administration [11]. In the present case, dexmedetomidine administration increased the power spectrum of delta waves, which may reflect slow delta oscillations. Conversely, no spindle in the alpha-wave region was observed, presumably due to the patient's deep sedation induced by the concurrent administration of midazolam [12]. Furthermore, dexmedetomidine administration reduced the power spectrum in the theta-wave region. The observed reduction in the theta-wave power spectrum might be owing to the synergistic effect of dexmedetomidine and midazolam [13], because midazolam is known to increase delta-wave power and decrease theta-wave power [14]. Behind this synergistic effect may be the involvement of different receptor targets, as midazolam acts on gamma-aminobutyric acid receptors and dexmedetomidine is a selective α_2_-adrenergic agonist. Moreover, the delta-wave power spectrum demonstrated a marked increase 60 minutes after administration of a muscle relaxant. This change may also be attributed to the increased effectiveness of midazolam owing to diminished physiological stress resulting from shivering cessation. Although the analysis of this case did not allow us to investigate the detailed causes of these EEG changes, further studies are warranted to explore the various EEG alterations induced by different drugs administered to suppress shivering, as well as those commonly employed as sedatives, such as midazolam.

Several methodologies for predicting neurological prognosis based on EEG emphasize frequency components. Some studies indicate that the power within the alpha-wave band serves as a reliable prognostic indicator [15,16]. However, as shown in this case, shivering noise in the alpha-wave band renders this factor unusable unless shivering is entirely absent. Conversely, the slow-wave region appears to be less affected by shivering. Therefore, focusing on the slow-wave region is logical, as it has been reported to be superior in predicting prognosis during the early post-resuscitation period [17]. Considering that muscle relaxants did not affect the slow-wave region in this patient, they may be deemed optimal for minimizing noise in EEG analysis. However, it should be noted that the effectiveness is limited by the fact that EMG activity can affect the slow-wave regions of the EEG [18]. Notably, dexmedetomidine may induce significant changes in slow-wave regions, as shown here. While noise reduction is crucial for EEG analysis, it is essential to understand both shivering noise characteristics and the effects of anti-shivering drugs on EEG.

NCSE occurs in 1-20% of patients post-resuscitation [19]. This patient did not present with NCSE, making it difficult to determine the effect of shivering noise on detection. Nevertheless, it is plausible that shivering-related noise could affect high-frequency regions, potentially obscuring epileptic EEG signals [20].

While the guidelines recognize the importance of EEG monitoring in post-resuscitation care, they offer limited discussion on the prevalence of shivering during TTM and the necessity to address it [3]. Furthermore, the guidelines do not specify the extent to which shivering affects EEG assessments [3]. Although it is not feasible to propose changes to resuscitation management protocols or guidelines based solely on this case report, managing shivering and understanding its impact on EEG interpretation are critical considerations in post-resuscitation care. We suggest that future research and guideline development should prioritize the optimization of shivering management strategies and the enhancement of EEG decoding techniques to mitigate shivering-induced artifacts.

## Conclusions

This case exemplifies the significance of shivering and its management with pharmaceutical interventions in the accuracy of EEG measurements. Given that shivering generates noise in the high-frequency region, prioritizing the slow-wave region for prognostic evaluation may be imperative. Conversely, it should be noted that anti-shivering medications can cause drug-dependent changes in the slow-wave region. It is crucial to recognize that these effects have the potential to compromise the accuracy of EEG-based neurological prognostication and detection of NCSE. To ensure accurate EEG readings, it is imperative to reliably cease any occurrence of shivering, as it may lead to erroneous interpretations. Further studies are warranted to evaluate the effects of shivering and anti-shivering agents on EEG monitoring during post-resuscitation care.
